# Retraction Note: Cellular immunotherapy using irradiated lung cancer cell vaccine co-expressing GM-CSF and IL-18 can induce significant antitumor effects

**DOI:** 10.1186/s12885-023-11123-7

**Published:** 2023-07-03

**Authors:** Hongwei Tian, Gang Shi, Guoyou Yang, Junfeng Zhang, Yiming Li, Tao Du, Jianzhou Wang, Fen Xu, Lin Cheng, Xiaomei Zhang, Lei Dai, Xiaolei Chen, Shuang Zhang, Yang Yang, Dechao Yu, Yuquan Wei, Hongxin Deng

**Affiliations:** State Key Laboratory of Biotherapy, West China Hospital, Sichuan University, Chengdu, Sichuan 610041 The People’s Republic of China

**Retraction Note: BMC Cancer 14, 48 (2014)**

10.1186/1471-2407-14-48

The Editors have retracted this article. After a correction was published in 2020 [1], additional concerns were raised about an apparent partial overlap between panels CD4-mIL-18 in Fig. 5B and CD8-mIL-27 in Fig. 6D in a previously-published paper by the same author group [2]. Another concern was the partial overlap between panels mGM-CSF and mIL-18 in Fig. 6A. The authors provided original images for Fig. 6a, but they did not address the image overlap. The Editors, therefore, have lost confidence in the integrity of this paper. The corresponding author has stated on behalf of all co-authors that they disagree to this retraction.
